# How Do Australians Manage Diagnostic Testing Risks? Focus Groups Linked to a Model of Behaviour Change

**DOI:** 10.1111/hex.70038

**Published:** 2024-10-02

**Authors:** Tomas Rozbroj, Catriona Parker, Romi Haas, Jason A. Wallis, Rachelle Buchbinder, Denise A. O'Connor

**Affiliations:** ^1^ School of Public Health and Preventive Medicine Monash University Melbourne Victoria Australia

**Keywords:** attitude, COM‐B, diagnostic test, overdiagnosis, public, qualitative, risk

## Abstract

**Background:**

Diagnostic tests carry significant risks, and communications are needed to help lay people consider these. The development of communications has been hindered by poor knowledge about how lay people understand and negotiate testing risks. We examined lay Australians' perceptions of diagnostic testing risks and how these risks are managed.

**Method:**

We completed 12 semistructured online focus groups with 61 Australian adults (18+) between April and June 2022. Participants were divided into younger/older (> 50 years) and male/female groups. Using semistructured discussion and exploring two hypothetical scenarios, we examined attitudes to diagnostic tests, their risks and how test risks were managed. Themes were identified, subanalysed to identify age and gender differences and mapped to the COM‐B model of behaviour change.

**Results:**

The six themes provided detailed accounts of how participants considered themselves able, empowered and assertive when negotiating testing risks and of complex ways in which relationships with health workers, personal experiences and structural factors influenced negotiating testing risks. COM‐B identified multiple opportunities for leveraging these lay beliefs in health promotion. It also identified barriers, including narrow concepts of testing risks, challenges during shared decision‐making and overestimation of personal influence on testing decisions.

**Significance:**

Our findings matter because they are a novel, detailed account of testing risk beliefs, linked to a model for behaviour change. This will directly inform development of test risk/benefit communications, which are a research priority.

**Public Contribution:**

The study design enabled participants to influence the discussion agenda, and they could comment on the analysis. Participants contributed insights about their needs, beliefs and experiences related to medical testing, and these will be used to shape future patient‐centred decision tools.

## 1 Background

All diagnostic tests and screening carry risks. Risks include overdiagnosis and subsequent unnecessary treatment, which may lead to adverse health outcomes, loss of quality of life, psychological burdens, costs and time off work [1]. Many testing decisions require patient understanding and deliberation [2] and come down to patients' value judgements and preferences [3]. For example, some modelling indicates a favourable balance between the harm and the benefit of mammography in Australia [4]. However, using mammography to gain a 15% reduction in breast cancer mortality increases its overtreatment by 30% [5]. Understanding these risks/benefits, and their own preferences and values, will help women negotiate mammography choices [6].

Lay people, patients [7] (recipients of health care [8]) and healthcare providers [9] tend to overestimate the benefits and underestimate the risks of diagnostic and screening tests. They find it challenging to imagine how tests could cause harms [10], are predisposed to test [11] and believe that they can avoid overdiagnosis and harmful therapies without foregoing the tests that precipitate them [12]. Some patients may understand the risks of medical tests insufficiently to provide ‘informed’ consent [13].

Shared decision‐making (SDM) prompts and awareness campaigns have been developed to help patients consider risks when facing diagnostic testing [14]. These may target specific testing scenarios [2], for example, decision aids about benefits and harms of prostate and breast cancer screenings [15] or general awareness, for example, in books [16, 17] and mass media strategies [18].

Existing tools and communications about testing risks have had limited impacts. The reasons are complex and multifactorial, including ingrained beliefs about the intrinsic value of testing, the complexity required to explain testing risks [19], inadequate communication from healthcare providers and significant vested interests promoting ineffective or harmful testing [20]. The extent to which lay people wish to be involved in avoiding testing risks remains poorly understood. The development of interventions directed towards them is based on attitudinal findings that they want engagement about test risks [12], and expert moral and epidemiological arguments about the benefits of patient‐centred tools to help them negotiate these [21]. Yet, we also know that few people worry about common testing risks [12]. For example, in computerised tomography scan referrals, participants seldom considered questioning doctors or worrying about risks [22]. Therefore, the extent to which lay people desire tools to help them avoid testing risks remains unclear. Assuming that they do wish to be involved, it is also unclear how they perceive and manage testing risks and what their gaps in understanding or management are. Therefore, we are unsure whether interventions actually meet their needs.

To address these gaps in the evidence, two bodies of work are needed: First, investigating lay peoples' beliefs, needs and preferences relating to diagnostic testing risks and their management. This will help inform intervention design, in particular message tailoring and framing. Therefore, studies geared towards intervention design, for example, linking individual experiences to models of behaviour, would be particularly valuable. The second body of work needs to focus on investigating the impact of these well‐designed interventions on real‐world behaviour.

In this study, we examined lay perceptions of diagnostic test risks and how these risks are managed, to inform such interventions.

## 2 Methods

We collected data using online semistructured focus groups with Australian adults. Data were analysed using thematic analysis and mapped to the capability, opportunity and motivation model of behaviour change (COM‐B) [23].

The study was reported according to the COnsolidated criteria for REporting Qualitative research checklist [24], included in Appendix 1.

All authors were involved in the study conception, refinement and interpretation of the results and in reporting. Data collection was completed by C.P. Thematic analysis was primarily completed by C.P. and T.R. COM‐B mapping was primarily completed by T.R. with input from D.A.O.C.

### 2.1 Design

The focus group design was chosen for several reasons. We required in‐depth data related to testing, and participants were likely to have heterogeneous testing experiences. Focus groups may capture a greater breadth of perspectives and more personal content compared to interviews, despite leading to similar themes capturing in‐depth insights [25]. Because testing risks could seem abstract to participants, we included fictitious scenarios to examine testing beliefs. Previous research demonstrated the usefulness of scenarios for generating open discussion in focus groups about screening risks [26]. These advantages outweighed limitations of focus groups, such as their tendency to generate ‘consensus’ views [27].

The focus group interview guide is included in Appendix 2. We used a semistructured web‐based (Zoom) design. Focus groups took approximately 90 min each and were video‐recorded. 15 min were dedicated to introductions, discussion of research ethics, administration and ‘ice breakers’ and 75 min were dedicated to exploring lay perceptions of diagnostic test risks and how these risks are managed. This comprised exploring factors influencing diagnostic testing choices; information needs about tests and testing risks; preferences, barriers and enablers regarding independently researching test risks and discussing risks in clinical consultations; and perceptions about low‐value testing. Participants were asked to consider two hypothetical scenarios designed to explore these topics within a relatable context [26]: (1) being offered a blood test at a general practice after presenting with fatigue and (2) a doctor resisting a patient request for an x‐ray for back pain.

The COM‐B framework of behaviour was chosen as an interpretative tool. It allowed us to link our findings to informing intervention design. It was chosen over other frameworks because it synthesises prior frameworks of behavioural interventions into a unified model, has been used widely and is supported by reliable evidence [28]. The COM‐B comprises three dimensions, subdivided into two subtypes each [23]. (1) Capability (C): subdivided into physical (e.g., disability affecting resource access) and psychological (e.g., ability to appraise resources). (2) Opportunity (O): subdivided into physical (e.g., having access to information) and social (e.g., accepting SDM). (3) Motivation (M), subdivided into reflective (e.g., conscious motivations) and automatic (e.g., subconscious drivers, biases) [29]. Identifying these factors helps explain behaviour (B) and how to influence it.

### 2.2 Participants and Sample

Individual focus groups comprised one of the following participant subgroups: younger women (aged 50 years or younger); younger men (aged 50 years or younger); older women (aged 51 years or older); and older men (aged 51 years or older). The subgroups were intended to detect important age‐ and gender‐related differences in managing testing decisions. In Australia, men and women are offered different diagnostic tests and treatments, and some screening programmes commence at age 50 [30].

We planned to initially conduct 12 focus groups in total: three per subgroup. Because most themes are discovered after three focus groups [31], we anticipated that 12 would enable us to reach saturation of broad, overall themes (e.g., extent to which participants self‐educate when considering tests). We recognised that participants would speak to diverse experiences with disparate tests, which would be unrealistic to capture in a ‘saturated’ way and also that saturation is a problematic concept in qualitative research [32], and studies must define it in relation to their aims. For us, broader beliefs were the focus.

We targeted between three and eight participants per group, which was appropriate for the online data collection modality [33]. All groups had the same interview guide. The focus group schedule was pilot‐tested before data collection.

### 2.3 Recruitment

We recruited focus group participants at the end of an online survey that investigated attitudes to diagnostic testing risks among Australian adults. Survey recruitment occurred between January and April 2022 using Meta (Facebook) advertising. We purposively sampled to include a diverse range of participant characteristics, including age, gender and state/territory of residence. Upon survey completion, respondents could register for focus groups. Eligible participants were then allocated to focus groups between April and June 2022, based on their availability. All focus group participants were offered A$40 gift vouchers.

### 2.4 Data Collection

Focus groups were completed between April and June 2022. A female, PhD qualified researcher who is experienced in qualitative research (C.P.) screened and allocated registrants and facilitated the groups. C.P. made research notes during the focus groups.

Written consent was required before participation. Participants had no prior relationship with C.P., and C.P.'s beliefs about the content or related characteristics were not discussed. At focus group commencement, participants were briefed about the aims of the study. At conclusion, they were offered an opportunity to view deidentified draft findings. Throughout, the facilitator monitored conversations, checking for verbal cues, dominance and halo effects [34], to proactively give all participants opportunities to share their perspectives. Most participants appeared comfortable sharing their perspectives, and transcripts showed that all made substantial contributions.

### 2.5 Analysis

Video recordings were audio‐transcribed by a professional service. Individuals were differentiated but not identified in the transcripts. Participants were not given transcripts for comment/correction.

Analysis comprised two stages. First, thematic analysis [35] was carried out to identify patterns in lay perceptions of diagnostic test risks and how these are managed. Codes were developed both deductively and inductively by C.P. and T.R. The structured parts of the interview guide, such as scenario questions, led to a few deductive codes (e.g., responses to Scenario 2). Most codes were developed inductively, through iterative reading and code generation. Coded data were monitored for internal homogeneity. Data were not analysed for intra‐group dynamics. Themes were refined and then analysed for data coverage by gender and age to identify differences relating to these two variables. Data saturation of broad themes was checked. Exemplary comments were selected, based on being judged to either represent the dominant sentiment in the theme; represent nuance that was important to understanding the theme; and represent contrary data that complexified the theme.

Second, we mapped themes onto the COM‐B to analyse the behavioural underpinnings of perceptions about diagnostic test risks and their management, and how strategies could influence them. We examined the C/O/M of the themes that we identified about B: how lay people understand and manage testing risks. We re‐analysed themes that had no subthemes as well as individual subthemes (but not their parent theme, to avoid duplication) to determine C/O/M domains. We focused on dominant narratives within themes (e.g., if the theme described participants' confidence in appraising testing risks). Following previous research [36], we differentiated whether C/O/M domains were explicitly evidenced (e.g., participants tended to state that they are confident information appraisers) or implied (e.g., participants described processes that suggested that they were confident information appraisers). This helps readers assess the strength of our claims. We also judged whether the C/O/M was a barrier or enabler for managing testing risks.

### 2.6 Ethics

This study was approved by the Cabrini Research Governance Office (07‐04‐11‐21) and the Monash University Human Research Ethics Committee (31176).

## 3 Results

The sample profile is presented in Table 1. Moreover, 295 people registered for the focus groups, 38% of the 774 who completed the survey. We completed 12 focus groups comprising 61 participants: 54% were women and the rest were men. No other genders participated. The mean [standard deviation, SD] age was 51 [18] years. Most participants lived in Victoria (48%) or New South Wales (21%). No participants withdrew, but some who agreed to participate did not attend. We collected over 15 h of data, which were coded into 62 codes and 7013 references. No new codes were identified after analysing six transcripts.

**Table 1:** Sample profile.

Focus group	*n*	Mean age	State/territory of residence							
			ACT	QLD	NSW	NT	SA	TAS	VIC	WA
1 Older W	8	68			3		1		4	
2 Older W	3	67			1				2	
3 Older W	6	71		1			1		4	
Older women	***17***	***69***		***1***	***4***		***2***		***10***	
4 Younger W	4	25						2	2	
5 Younger W	7	34		1	2				3	1
6 Younger W	5	39	2						1	2
Younger women	***16***	***34***	***2***	***1***	***2***			***2***	***6***	***3***
7 Older M	5	69			1		1		3	
8 Older M	4	71			1				3	
9 Older M	5	66			3				1	1
Older men	***14***	***69***			***5***		***1***		***7***	***1***
10 Younger M	6	33		1	2		1		2	
11 Younger M	3	34		1					2	
12 Younger M	5	31		1				1	2	1
Younger men	***14***	***32***		***3***	***2***		***1***	***1***	***6***	***1***
Sample	**61**	**51**	**2**	**5**	**13**	**0**	**4**	**3**	**29**	**5**

*Note:* Bold, italicised text indicates subtotals. Bold unitalicised text indicates sample total.

### 3.1 Thematic Results

We identified six main themes. Two of these each had two subthemes. Exemplary comments are presented in Table 2.

**Table 2:** Participant quotes.

ID	Group	Quote	Theme/s^a^^,^ b
1	Younger women	I feel quite capable, I like to be discerning of resources, I like to see what's out there. I do actually like to look at different opinions and things like that that I like to weigh it up with other sources that I know are credible. I feel quite confident in doing that, but I know some people can go down a rabbit hole and ignore the credible information as well. But I like to have all different opinions and weigh it up myself.	3.1.1.1. Proficient information appraisers.
2	Older women	There are areas of medicine where most doctors have not received training. I know in health pathways, I don't know how many people are familiar with health pathways, but they're coming as a main source of information for primary care practitioners. And some Primary Health Networks have got like 800 health pathways. Your one doctor is not going to be fully familiar with 800 different pathways. And those pathways include diagnostic tests as well as other things. So, if you have a condition which is either not common or not taught in universities, not well known, then you do need to actually bring to your doctor here's what has been recommended by this group of experts at this university or say at John Hopkins in the United States. So sometimes you actually do have to take responsibility for educating your doctor. And then you're say here's… It's not me saying, oh, I want you to test for this, but saying, here's this information that will inform you and now can you please do what it says.	3.1.1.1. Proficient information appraisers; 3.1.4. Relationships w/doctors impact testing choices; 3.1.5. Importance of asserting needs/preferences.
3	Older men	[Blood test scenario] I would feel unsatisfied apart from the fact that he didn't examine me, for instance, when there's lots of reasons why, for instance, when you've had sleep and when you wake up. I think that as [another participant] said that one of the top things that you would think of would be sleep apnoea. So, you would be asking questions along those lines to begin with, even before thinking about a blood test. There aren't that many things that you'd pick up with a blood test to indicate why you're feeling tired in the morning.	3.1.1.2. Clinical judgements about testing scenarios; 3.1.3.2. Make testing decisions with patients.
4	Older women	[Back pain scenario] From that scenario I would have thought a couple of visits to the physiotherapist would have been suggested…. I mean unless you've just tried to lift 100 kilos and put your back out the only outcome you're really going to have anyway is go to a physiotherapist.	3.1.1.2. Clinical judgements about testing scenarios.
5	Older women	My cancer would have been picked up a lot sooner if I'd had an ultrasound instead of a mammogram because I told them that my family's cancer is only being picked up by ultrasound and they just ignored me. So, they would have picked it up a lot sooner had they done the ultrasound.	3.1.2. Interpreting testing risks through prism of experience.
6	Younger women	[in response to LBP scenario] My husband was also diagnosed for, I forgot the word for that, but then he trusted his health professional very badly, GP. And then they asked him to get something like a day surgery or something under the effect of anaesthesia. So, I just told him, why don't you consult another GP. So, he consulted, and the GP said, no, the diagnostic test is not necessary because you are in that normal range. And the thing was because there was one GP who said yes and one for no, and because he works in the medical school, I told him, why don't you check with the Head of Department of radiology. So, he had a conversation, and then the department said it's not needed. So, he took the same feedback to his GP and the GP was like, I know you can easily manage with diet, but we just wanted to make sure… he wanted to know more, that's why he used my husband as a guinea pig. I always have a feeling, that if you have a very simple test like blood tests and all, that's fine, you can go for it. But if there are complex tests, you surely need to take a second opinion.	3.1.2. Interpreting testing risks through prism of experience; 3.1.4. Relationships w/doctors impact testing choices; b3.1.4. Relationships w/doctors impact testing choices; 3.1.5. Importance of asserting needs/preferences.
7	Older men	What's the purpose of this, what is the outcome, given the results what's the treatment going to be, what is the risks associated with some of these tests. And of course, in some cases what are the costs of these tests, because not everything is covered by Medicare. And it's mainly around the question is what do you expect to get from this test, what are we going to do about it we get those results, is there something I can do in the meantime that may improve this and then let's go have the test.	3.1.3.1. Participants sought to understand risks and benefits; 3.1.3.2. Make testing decision; 3.1.6. Structural factors influencing testing choices.
8	Younger women	[One participant] I wonder if the risks that we're prepared to take for our own health are different to what we're prepared to take for our kids. I don't know, I know with my own kids I think a lot more about what are the risks of any treatment? And do the benefits outweigh those risks or potential harms? There seems to be somehow so much more at stake. [Another participant] It's different, as well, for your kids because you have to do that risk, benefit weigh up for them, on their behalf… You're thinking… what will this mean for them in the long term… I do feel like there's a different level, whereas I don't think we'd think twice about certain things for myself.	b3.1.3.1. Participants sought to understand risks and benefits; 3.1.5. Importance of asserting needs/preferences.
9	Younger men	I know there's also some concerns about people who do regular testing when it's not necessary. Particularly when they're chasing no symptoms and don't really have risk factors. The problem is that if you do a major scan like an MRI, any little marking or abnormality, or fleck on the scan could be interpreted in a lot of different ways. Now, if you've gone in because you say I've got these terrible pains in my stomach or my leg really hurts, and sure enough they find some abnormality in the stomach or the leg, then you probably have got something going on there. But if you're just looking at the mark with no symptoms whatsoever, it can be hard to say what's disease and what's people being different.	b3.1.3.1. Participants sought to understand risks and benefits.
10	Older women	The industry has got the best name ever: ‘Healthcare’. Because there is the word ‘care’ at the bottom of it, people trust. And look, that's okay. Everybody is in the business to make money, not really to care. And that's fine. So, it took me a while to understand what were the tests marketed by the industry? It's very complex.	b3.1.3.1. Participants sought to understand risks and benefits. b3.1.4. Relationships w/doctors impact testing choices.
11	Older men	To figure out what tests are worth doing: a) you have to know what you're looking for, and b) you have to know what you're going to do once you get the answer.	3.1.3.2. Make testing decisions with patients.
12	Older women	It's probably about taking some responsibility for having knowledge of what you're having done or what might be wrong with you, or I guess not just blindly following your health professional. But then, as [another participant] said, if something's wrong with my roof and I have someone in to fix it, I don't know anything about roofs. So, I'm going to trust them to diagnose the problem, test it and fix it. And I guess, should we not have that expectation in our medical professionals as well? Well, yes, we should. I don't ask the roof guy much. It's difficult. And clearly, your own health is way more important than your roof.	3.1.3.2. Make testing decisions with patients; 3.1.5. Importance of asserting needs/preferences.
13	Younger men	I guess to some degree if there's trust you're more likely to ask questions. But I guess also if there's trust you might just go, well, fair enough and walk out and have the needle put in your arm.	3.1.4. Relationships w/doctors impact testing choices.
14	Younger men	[One participant] It's about a conversation. And I think if you're going into the conversation with your doctor and say, I'd like you to order these tests for me now, then it's very presumptuous. [Another participant] To second that, doctors are not vending machines for whatever health outcomes you want. They've done the study. They know more than us. Obviously, there might be situations where a doctor might not be listening to you, but at that point, you would just go get a second opinion. If a different doctor is saying the same thing, then maybe you just have to accept that you don't know as much as you thought you did.	b3.1.1. Participants considered themselves to be informed patients; 3.1.3.2. Make testing decisions with patients; 3.1.4. Relationships w/doctors impact testing choices; b3.1.5. Importance of asserting needs/preferences.
15	Younger women	[in response to LBP scenario] I would probably ask the doctor, at what point would you do a test? What factors would make you concerned enough to do a test if this is not enough? Maybe they say, it seems like you haven't had it for long enough or your pain is being managed, or something like that. Then I can decide if that's a good enough reason and like the others, go find, maybe, somewhere else as well.	3.1.5. Importance of asserting needs/preferences.
16	Older women	I would always want to weigh benefit from costs. So, for me, I'd think much more seriously about an invasive testing and tests that have a higher risk factor. And also, I assess the doctor who's prescribing the test. Because sometimes you'll get a doctor who is wanting to cover themselves and they'll order everything they can think of and that doesn't impress me. I'd much rather a doctor that's thorough in their questioning, in their examination and then talk through testing relevant to the symptoms and them to share what the goal of any testing is. Again, because I've got to go 300 kilometres to [country town] to get any significant testing done other than blood tests. So, it's got to be not trivial but within context worthwhile.	3.1.3.1. Participants sought to understand risks and benefits; 3.1.4. Relationships w/doctors impact testing choices; 3.1.6. Structural factors influencing testing choices.
17	Younger women	I'd definitely say time. So, if I felt like they were like, okay, is that all today, here's your paper, see you, out the door. If I had had to wait for my appointment and I knew there was a full waiting room out there, I would feel then if I ask questions, I'm just pushing everyone back and contributing to that. I think I find it difficult, it's really unfair on GPs in a sense, but when they, it's such a power dynamic. So, they're sitting behind a desk, typing away, maybe not even making eye contact with me. So, it's not really an inviting place for me to ask a question. So, okay, you clearly think this is the right thing for me to do. You're not even really looking at me, I'm out, I'll just get this done. It's a lot about the manner.	3.1.4. Relationships w/doctors impact testing choices; 3.1.6. Structural factors influencing testing choices.

^a^ Single comments could be coded to multiple themes.

^b^ Quote that contradicts a theme and/or presents data that were insufficient to form a theme.

#### 3.1.1 Participants Considered Themselves to be Informed Patients

Most participants described having high confidence in their abilities to identify and independently appraise information about testing choices and risks (Section 3.1.1.1). This confidence also appeared to manifest when participants sought to make clinical judgements about the focus group testing scenarios (Section 3.1.1.2).

##### 3.1.1.1 Proficient Information Appraisers

Participants typically described sophisticated ways in which they independently find, select, appraise, comprehend and use resources to understand testing choices and risks. Some also discussed strategies to avoid poor‐quality information, for example, citing which websites they considered reputable (e.g., Mayo Clinic and Better Health Channel). Some participants also described using peer‐reviewed articles. The main challenges described by informed participants about appraising testing choice and risk information were comprehending jargon and highly technical resources.

In several focus groups, participants reflected that they were particularly informed and may be atypical compared to the average person. They were concerned about how other, less effective information appraisers would handle self‐directed research about testing choices and risks and whether they would be susceptible to conspiracies or other non‐evidence–based health information. Individuals across focus groups also discussed the limitations of self‐directed research and their reliance on clinicians to make testing decisions (e.g., exemplary comments 12 and 14).

##### 3.1.1.2 Clinical Judgements About Testing Scenarios

We asked participants how they would handle two hypothetical scenarios (being offered a blood test after presenting with fatigue; doctor resisting a patient request for an x‐ray for back pain). We did not ask them to guess the causes of the hypothetical ailments.

A surprising number of participants nevertheless tried to guess the causes. For example, regarding the blood test scenario, they commonly guessed that either sleep apnoea or low iron levels were responsible for the fatigue. The assumptions that they made about the cause of the fatigue then led some to judge the appropriateness of diagnostic testing in the scenario and informed their decisions about what information they wanted. For example, if low iron levels were suspected, they thought that a blood test was appropriate; if sleep apnoea was suspected, participants may have become perplexed about why a blood test would be requested. Those suspecting sleep apnoea might have questioned the risks and necessity of the blood test.

#### 3.1.2 Interpreting Testing Risks Through the Prism of Experience

We asked participants broad questions that related to testing choices and risks, such as “is there such a thing as ‘too much information’?”. Participants were asked to keep their answers general in nature and were reminded that they do not need to provide medical details about themselves or others. However, many handled these questions by relating them back to personal experiences, including medical scares, unnecessary testing and serious illnesses. The lens of experiences through which they considered our scenarios appeared to shape their conclusions. For example, when assessing the benefits/harms of avoiding testing, less favourable responses were linked to descriptions of experiences of diseases that were diagnosed late. More favourable responses were linked to experiences of unnecessary medical encounters resulting from a false‐positive test result.

#### 3.1.3 Information Needs From Healthcare Consultations When Facing Testing Choices

Participants described their information needs when facing testing choices and considering risks. Two subthemes emerged: capturing the importance of understanding the anticipated risks and benefits of tests (Section 3.1.3.1) and seeking to understanding the broader rationale for testing choices (Section 3.1.3.2). Most participants sought information from doctors during consultations as one part of a broader information‐seeking pathway that also included self‐directed research before and after consultations.

##### 3.1.3.1 Participants Sought to Understand Risks and Benefits

Almost universally, participants focused on perceived risks and benefits when considering tests. Commonly, perceived risks were direct physical consequences from tests, such as pain, complications and discomfort caused by the test. These perceived risks were balanced against perceived benefits. Commonly, perceived benefits were understanding ones' ailment and healthiness, ‘catching disease early’ and beliefs that testing was required for initiating a desired treatment pathway. Potential risks and benefits were considered in relation to family history and perceived life stage. For example, some older participants were disinclined to test for certain complaints that they considered to be part of getting old, such as pain, and were motivated to undertake screening for age‐related risk factors, for example, monitoring blood glucose levels.

Overall, participants had positive sentiments towards tests and had a limited sense of potential testing harms. They seldom mentioned false‐positives, overdiagnosis or the potential downsides of knowing about a benign condition or about a condition for which there is no known treatment or cure. Data on overtesting and overdiagnosis were insufficient to constitute a theme. However, across the focus groups, there appeared to be some understanding of unnecessary testing, and particularly of defensive medicine practices and problematic incentives for testing (such as profit for test providers). A few times, participants mentioned being more careful when making testing decisions for dependent children.

##### 3.1.3.2 Make Testing Decisions With Patients

Participants expressed diverse preferences for involvement in their testing choices, including involvement in negotiating testing risks. Some preferred to leave testing decisions almost entirely to their doctors, reasoning that the doctor knows best. Most wanted at least some personal involvement, particularly to understand the rationale for testing recommendations (as well as potential risks and benefits). Participants sought to feel in control, which involved knowing what test will happen, why and what it will involve. A minority of participants aimed to be sufficiently informed to take control of judging whether a test was right for them. When presented with onerous tests (including in terms of time commitment, discomfort, perceived risk) or unfamiliar tests, participants were likelier to want more information about these tests and/or greater involvement in decision‐making. However, they also reported feeling less able to participate in decision‐making about unfamiliar tests.

#### 3.1.4 Relationships With Doctors Impact Testing Choices in Complex Ways

Although participants described requesting certain diagnostic tests, most testing choices started with a doctor's recommendation. The extent to which participants trusted their doctor influenced how they would respond to that recommendation. Participants described ways in which a trusting relationship gave them confidence to ask questions about risks. However, these participants also seemed less likely to think that asking questions about risks was important, because they had faith in the decision being made for them. Most participants described positive relationships with their doctors. However, a lack of trust was implied in some comments. For example, participants expressed concerns about financial incentives driving doctors' testing recommendations or reservations about the competency of some doctors. These perceptions seemed to prompt participants to question the testing recommendation and associated risks more.

#### 3.1.5 Importance of Asserting Needs and Preferences in Testing Decisions

Typically, participants considered themselves capable and engaged in SDM about tests, including testing risks. Most believed that they were largely able to self‐advocate in testing decisions during clinical consultations, albeit with limitations.

In the scenario where a doctor resisted a patient request for an x‐ray for back pain, participants tended to have unfavourable responses to the doctor's decision. Some explained that they would try to understand why the doctor was resisting their wish to have an x‐ray and judge whether this resistance was reasonable. However, if the explanation for resisting was deemed inadequate, for some participants, this would potentially damage the doctor–patient relationship and could prompt them to seek a second opinion or change doctors. Other participants said that they would accept and trust the doctor's decision.

Some of these responses exposed participants' boundaries between recognising the role of experts and apparent willingness to question or even reject expertise to receive the health care that they believed they needed. However, when participants described past healthcare encounters rather than the hypothesised ‘x‐ray for back pain’ focus group scenario, it appeared that they commonly did what doctors recommended.

#### 3.1.6 Structural Factors Influencing Testing Choices

Participants described diverse structural factors influencing how they negotiated testing choices and risks. Four factors were common. First, costs of tests. Tests with high out‐of‐pocket costs prompted many participants to ask more questions about their risks, benefits and necessity and waver about proceeding with the test. Tests that were cheap or free were seldom perceived to require deliberation unless they were considered risky, although several participants did mention not wishing to waste public resources on unnecessary tests. Second, frustration with repeated tests, such as when a test was requested by a general practitioner and again by a specialist following referral. Third, perceived time poverty of doctors and short appointment times were barriers to engaging in SDM about testing and test risks. Finally, accessibility was an issue for some participants. This included needing to travel for tests or finding time to attend appointments, particularly among participants who described living remotely. Conversely, in the blood test scenario, we mentioned that the pathology nurse was on‐site and this contributed to some participants deciding to say that they would have the blood test.

### 3.2 Age and Gender Differences in Thematic Results

Themes were fairly consistent across age and gender groups. Younger participants were likelier than older ones to describe their personal capacity to research testing choices and risks. They more commonly described being informed patients (Section 3.1.1) and having information appraisal proficiency (Sections 3.1.1, 3.1.1.1). Younger participants were also likelier to bring up their information needs when faced with testing decisions (Section 3.1.3) and desire to be involved in decision‐making about testing and test risks (Sections 3.1.1, 3.1.1.2).

Older participants were likelier to discuss interpreting testing risks through the prism of their experiences (Section 3.1.2), as were men compared to women, regardless of age.

Men were likelier to discuss their information needs from healthcare consultations when facing testing choices (Section 3.1.3). Women emphasised more than men the importance of asserting their needs and preferences in testing decisions (Section 3.1.5) and described structural factors in their testing decisions more often in the focus groups (Section 3.1.6).

### 3.3 COM‐B

Themes mapped onto COM‐B are displayed in Table 3, and explanations for the judgements are included in Appendix 3. Most themes/subthemes directly related to motivation (*n* = 5) and capability (*n* = 4); two related to opportunity. Participant comments suggested high psychological capabilities and reflective motivations to negotiate testing choices and risks, and seldom described barriers to doing so. However, our analysis suggested that participant confidence and aspects of their clinical SDM may be barriers to understanding and managing testing risks. Several themes described both enablers/barriers or whether it was a barrier/enabler depended on other factors (e.g., contextual, individual).

**Table 3:** Themes mapped to the COM‐B model.

Theme	Which COM‐B domain does theme map to?^a^, ^b^						For managing test risks, is it? Enabler/barrier/both/depends
	C: Phy.	C: Psy.	O: Phs.	O: Soc.	M: Ref.	M: Aut.	
3.1.1.1. Proficient information appraisers.	✓	✓	✓		✓		Enabler
3.1.1.2. Clinical judgements about testing scenarios.	✓	✓			✓	✓	Both/depends
3.1.2. Interpreting testing risks through the prism of experience.	✓	✓			✓	✓	Either
3.1.3.1. Participants sought to understand risks and benefits.		✓	✓	✓	✓	✓	Depends
3.1.3.2. Make testing decisions with patients.		✓	✓	✓	✓	✓	Enabler
3.1.4. Relationships w/doctors impact testing choices		✓	✓	✓	✓	✓	Depends
3.1.5. Importance of asserting needs/preferences	✓	✓		✓	✓	✓	Both
3.1.6. Structural factors influencing testing choices.	✓		✓		✓		Depends

^a^ Dark shading = direct evidence of COM‐B domain in theme; light shading = implied evidence of COM‐B domain in theme.

^b^ COM‐B refers to capability, opportunity and motivation and their influence on behaviour. Each of C, O and M has two subdomains. C: physical and psychological; O: physical and social; M: reflective and automatic.

## 4 Discussion

From 12 focus groups with 61 Australian adults, we identified six main themes and four subthemes capturing perceptions of diagnostic test risks and how these are managed. The study was conceived amongst a relative consensus in the field that we need patient‐centred or patient‐led approaches to reduce testing‐related harms [20, 37]. Our discussion examines implications for developing such approaches, summarised in Table 4.

**Table 4:** Summary of implications for patient‐centred strategies to reduce testing risks.

1.	Knowledge‐based interventions may have limited impact but remain crucial
2.	Help patients to speak up during clinical encounters
3.	Explaining risks to lay people should reference empowerment where appropriate
4.	Unfamiliar tests and new ailments present opportunities for targeted engagement
5.	Reducing access and increasing costs for low‐value or harmful tests should be considered
6.	Multipronged strategies to address testing risks, particularly influencing clinicians, remain key
7.	No one size fits all

### 4.1 Many Feel Empowered and Capable to Negotiate Testing Risks, but Few Understand Them

When negotiating testing choices, participants typically felt able and motivated to undertake self‐directed research, discuss risks and benefits in clinical encounters and assert their preferences. Many believed that they have already been scrutinising the risks of diagnostic testing and understood these regarding routine tests (Sections 3.1.1, 3.1.3, 3.1.5; COM‐B results). However, most participants had a limited sense of testing harms, such as overtesting, overdiagnosis or overtreatment (Section 3.1.3.1). Similarly, previous research has found that lay people have low awareness of testing risks [12], misappraise the relative risks and benefits of particular tests [7, 19], are confident in their ability to use information to avoid testing risks and are keen to be engaged and involved in doing so [12].

Current knowledge points to limited opportunities to reduce testing risks by addressing knowledge gaps about testing risks. On the one hand, the apparent motivation and empowerment of lay people to self‐manage testing risks may present opportunities to engage them with educational interventions. Some evidence supports this. Consumer‐ and patient‐led initiatives have curbed the provision of unnecessary and risky health care [38], and instances of individuals pushing back against potential overdiagnoses are documented [39]. Understanding ‘overtesting’ empowers people to avoid it [12], and shifting risk perceptions influences health behaviour [40]. Patient‐centred strategies also influence doctor behaviour [41]. On the other hand, broader harms of testing, such as overtesting, are particularly challenging to explain [12]. Favourable attitudes to testing may drive cognitive dissonance about testing risk messages and undermine their impact [19]. Even when people accept that clinical harms outweigh benefits, they may favour testing due to other perceived benefits, such as it signifying that their well‐being is cared for [42].

Overall, the impact of knowledge‐based interventions about testing risks is unclear, but probably modest. Nevertheless, they may form a valuable part of multipronged strategies, including behaviourally informed interventions for lay people. Irrespective of impact, explaining facts as best as possible is integral to seeking informed consent and in SDM. Therefore, *Knowledge‐based interventions may have limited impact but remain crucial*.

### 4.2 Patients Feel in Control, but Doctors Decide

Participants largely seemed to follow doctors' advice about tests (Section 3.1.4; COM‐B), despite feeling that they influenced testing choices (Section 3.1.5). Trust in doctors promoted SDM but reduced motivation to question risks, whereas distrust inhibited SDM but motivated greater vigilance about test risks (Section 3.1.4). The juxtaposition of high perceived and desired control with largely appearing to do what doctors suggested fits with a psychological insights that people tend to feel more in control of decisions than they actually are [43]. However, it also appeared that participants' self‐efficacy and self‐advocacy in negotiating testing decisions were diminished once they were in the clinic.

We draw two implications. First, patient‐centred strategies should only be expected to have a modest impact on addressing inappropriate testing and test risks. Other levers are needed. Multipronged strategies to address testing risks, particularly influencing clinicians, remain key. Doctors largely drive testing decisions and frequently order low‐value tests [44]. Lay people have a limited role in medical decision‐making, and wish to be involved in it to varying extents [45]. Some interventions targeting doctors have reduced excessive testing [46], and other research examining doctor‐led strategies to optimise test requests is ongoing [47].

Second, patient‐centred strategies nevertheless remain important and require optimisation. Our results suggest that one area for optimisation is helping patients to speak up during clinical encounters. This may align with patient preferences; many want to be involved in testing decisions [48, 49] and want tools that support this [22]. Optimising decision aids addressing testing decisions remains important. Presently, these are overwhelmingly informative rather than persuasive about test risks [2]. Perhaps persuasive tools are needed to help patients consider testing risks in a broader way.

### 4.3 Framing Messages Around Empowerment

Participants overwhelmingly emphasised their empowerment and capability to understand (Section 3.1.3.2) and actively negotiate testing choices (Sections 3.1.1, 3.1.5; COM‐B), whilst being guided by experts. The importance of empowerment in lay testing choices was also identified elsewhere [42], and a large body of work applying self‐determination theory suggests that the need for autonomy drives health behaviours [50]. In contrast, despite calls for empowering patients to avoid the risks of low‐value care [51], some messages seeking to do so have been interpreted as disempowering by patients [12]. For example, claims that a test will cause them to choose low‐value treatments, or that it is better ‘not to know’, may be interpreted as disempowering [12]. Other framing, such as knowledge about risks empowering choice and helping patients stay in control, should be explored. Importantly, information about low‐value care and overdiagnosis can itself be disempowering [52], and careful evaluation of strategies is needed. Nevertheless, explaining risks to lay people should reference empowerment where appropriate.

### 4.4 Risk Assessment Depends on the Test, Who It Is for and Costs

Participants were usually unconcerned about the risks of common and unintrusive tests (Scenario discussions; Section 3.1.2) or inexpensive tests (Section 3.1.6). Expensive, unfamiliar, intrusive or intimidating tests, especially for dependents, prompted metacognition about risks. However, some participants felt less empowered negotiating these relative to when facing familiar tests and may require more support. Experiences shape the interpretation of testing risks [19]. Both deliberative (deliberative, rational) and affective (emotional) responses shape risk perceptions [40]. Therefore, unfamiliar tests and new ailments present opportunities for targeted engagement. Furthermore, reducing access and increasing costs for low‐value or harmful tests should be considered.

### 4.5 No One Size Fits All

We found no universal formula for explaining testing risks to lay people. This was evident in the diversity of opinions and experiences captured in our thematic results and in the barriers/enabler analysis of COM‐B showing that many themes could either/both help or hinder strategies. Consequently, our discussion calls for tailored interventions to help lay people negotiate unfamiliar tests, and highlights the potential for overdiagnosis messaging to either empower or disempower lay people, depending on circumstances. Therefore, although our findings provide insights about how lay people perceive and negotiate testing risks, and how strategies can support them, no one size fits all.

### 4.6 Strengths and Limitations

Our study had notable strengths. The online modality enabled us to include participants in remote parts of Australia, and our sampling strategy allowed us to scrutinise age and gender differences (albeit within the limitations of a qualitative design). The use of two testing risk scenarios increased the granularity of our data and may have promoted open discussion [26]. The use of the COM‐B model enabled us to direct our findings towards identifying patient‐centred strategies to address testing risks, which is a research priority [53]. The study fills an important research gap in a timely topic.

Our study also had notable weaknesses. The sample self‐selected into the pool of registrants, likely out of interest in the topic. They did so following a survey about testing risks and benefits. This may have primed them, although significant time passed between survey completion and focus groups, and the survey was not mentioned by any participants during focus groups. The sample considered themselves well informed and engaged, although we could not verify this. If these self‐perceptions were accurate, some findings, particularly around self‐efficacy, may poorly reflect the experiences of less informed and less engaged Australians. During the focus groups, it turned out that several participants had healthcare training. It is possible that the views of participants with expertise may have disproportionately influenced how other participants in their group engaged with the content. However, we detected no evidence of this. The facilitator was careful to manage discussions and focus group participants appeared comfortable sharing their perspectives.

## 5 Conclusions

Communicating about testing risks and benefits to lay people is important, but it may have limited impact as a patient‐led strategy to help lay people avoid risks. Opportunities for engagement exist in empowering lay people, particularly to better self‐advocate during clinical encounters and when facing unfamiliar, expensive or invasive tests. Our findings underscore the importance of the vast body of ongoing work to support shared decision‐making in clinical encounters, in conjunction with strategies to support healthcare worker‐ and policy‐level changes to reduce risky and low‐value diagnostic test use.

## Author Contributions

**Tomas Rozbroj:** conceptualisation; investigation; funding acquisition; writing–original draft; methodology; writing–review and editing; formal analysis; project administration; and supervision. **Catriona Parker:** conceptualisation; methodology; project administration; investigation; writing–original draft; and formal analysis. **Romi Haas:** conceptualisation; writing–review and editing; and funding acquisition. **Jason A. Wallis:** conceptualisation; funding acquisition; and writing–review and editing. **Rachelle Buchbinder:** conceptualisation; funding acquisition; writing–review and editing; supervision; and resources. **Denise A. O'Connor:** conceptualisation; funding acquisition; supervision; and resources.

## Conflicts of Interest

The authors declare no conflicts of interest.

## Supporting information

Supporting information.

Supporting information.

Supporting information.

## Data Availability

Research data are not shared.
