# Supplementary material for: How Do Australians Manage Diagnostic Testing Risks? Focus Groups Linked to a Model of Behaviour Change
Source: Health Expect. 2024 Oct 2;27(5):e70038. doi: 10.1111/hex.70038 (PMC11447086; doi:10.1111/hex.70038)
Supplement: Supplementary file 2 — Supporting information. [file HEX-27-e70038-s003.docx]

# Appendix 2: focus group schedule

**Introduction script**

**Thanks** to everyone for coming along. My name is Cat and I’m a researcher with Monash University and Cabrini Health. <if other moderator is present introduce here> I just want to check that everyone has read the participant information statement? Does anyone have any questions?

**What’s this about**

This is a research project about ensuring Australian’s get the right amount of healthcare. Sometimes they miss out or are delayed getting the care they should have. For example, there can be excessive hospital waiting lists for some procedures like hip replacements.

Other times, people get unnecessary care that they don’t really need. For example, there are some treatments that have been proven not to work but are still offered to people. It’s easy to imagine then that people getting unnecessary care are contributing to the waiting lists and they could also be harmed by care they don’t need.

Therefore, there’s a need to find out how to ensure that Australians get just the right amount of healthcare.

Identifying the solutions is complex and the activity/research you are a part of today is about working with the public to understand what kind of care people expect, how they perceive the roles of tests and diagnoses, and what kind of roles, if any, the public should play in reducing unnecessary tests and medical care.

Any questions about this?

**Why are you here** When we do focus groups for research we usually do them face-to-face but today we’re doing them virtually. I expect our time together will be about one and a half hours. Today’s session is being recorded so I can engage with you rather than scribbling down notes. I do want to remind you that you can withdraw at any time and even withdraw anything you said for up to 4 weeks after today. It‘s a different experience in a virtual environment and can be easier for people if cameras are on - however the decision is entirely yours. I do just need to remind you, that we will record what is on the screen and the audio and even if your camera is off, we still record audio.

**Rules;** I ask that everyone is respectful of one another’s opinions, beliefs and experiences - understand that they might be different to your own. And if they are, we’re interested in that - tell us about why you disagree, but do it in a respectful way. Try not to talk over one another. You can use the technology to raise your hand or wait for a natural pause in the conversation to start talking.

It’s best to have other technology e.g. mobile phones, facebook, email etc. switched off or on mute to reduce distractions. We operate under a principle of Chatham Rules which is similar to ‘what happens in Vegas stays in Vegas’. What we mean by that is, this is a safe space, a confidential space - what you hear in here shouldn’t be discussed elsewhere out of respect for one another. Finally, it shouldn’t need to be said, but racism, name-calling, swearing, uncouth, obtrusive or offensive behaviour will not be tolerated and we reserve the right to exit such individuals from the focus group without explanation or warning. Any questions so far?

**Icebreaker**

Which state do you live and your favourite things for fun or relaxation

Now we all know each other a little better.

**Online tools**

There are some tools I want to show you how to use that can enhance your participation today and I may ask you intermittently to use them too.

<name display change>

<take through emojis>

<raising your hand>

<take through chat> (limited to only with the moderator)

Sometimes there are technology challenges so please be patient with one another. If you drop out, don’t panic, dial back in if you can. Don’t be intimidated by the fact that I may call on you specifically to speak. It’s ok if you don’t have anything to add to the conversation. I just want to make sure everyone has an opportunity to speak and be heard.

You’ll hear a term ‘diagnostic testing’ a lot over the next hour so I just want to let you know what I mean by that.

**Diagnostic testing**: a test we use to rule out or confirm the presence of a disease. Usually undertaken in response to symptoms. Things like blood tests, an x-ray, a biopsy etc.

And of course, please ask me during the questions if you’re not sure of the meaning of any of the words I use.

Does anyone have any questions?

**General questions**

1. What reasons might you decide to have a diagnostic test? (expect doctor tells me)
2. PROMPT are there any situations you can imagine where you wouldn’t have a diagnostic test your doctor has suggested? Please describe the situation.
3. How important is it for patients to think about whether or not they should have the diagnostic tests their doctor has suggested?
4. PROMPT what assumptions (things we accept as truth) do patients make about the diagnostic tests they get? (expect: will give a diagnosis)
5. Thinking back to past doctor visits how likely (or not) are you to ask the doctor questions about the diagnostic test they’re suggesting you have?
6. PROMPT if likely, what sorts of things do you ask? Why do you ask questions in the first place?
7. PROMPT if unlikely, why not? Probe for information about the interaction, environment etc.
8. When your doctor orders diagnostic tests what type of information do you expect them to tell you?
9. PROMPT is there such a thing as ‘too much information’? This is information you don’t need or don’t want to know. Why/how/what would be better?
10. Explain to me how you decide whether or not you should have a diagnostic test your doctor is suggesting? What things do you consider or process you use? (expect doctor tells me, want answer to my symptom)
11. PROMPT for if doctor tells me to: Are there any situations where you might not immediately follow what the doctor suggests? What are they?
12. PROMPT do you consider the risks (harms) and benefits into your decision-making process? How/in what way?
13. PROMPT How do you work out what are the risks (harms)? How do you work out what are the benefits?
14. PROMPT What harms might there be from diagnostic testing?
15. Do you think it’s possible you or someone you know has had unnecessary diagnostic tests in the past? These are ones they didn’t really need. In your response you don’t need to provide specific medical information about yourself or someone you know.
16. PROMPT if yes, how does it make you feel? Does it matter? Should it have been avoided? How do you think it could have been avoided?
17. PROMPT if no, what makes you feel this way? E.g. faith in doctors, own research etc
18. How often do you think people do their own research about the diagnostic tests the doctor orders?
19. PROMPT how accurate is the information that people find from doing their own research?

If accurate - what factors contribute to obtaining accurate info e.g. credible websites

If not accurate - what factors contribute to obtaining non-accurate info e.g. using forums

1. Are there any downsides to patients doing research about diagnostic tests that a doctor suggests?
2. PROMPT How equipped or capable do you feel to do your own research?
3. PROMPT What makes it harder to do your own research? Probe for life things or don’t understand when I’ve looked before
4. PROMPT What other things influence whether people do their own research about diagnostic tests?
5. PROMPT for those haven’t done any research about diagnostic tests, what are some of the reasons for this?
6. In the past if you have done your own research about diagnostic tests what has been challenging when you’re undertaking your own research? Probe for comprehension

<STOP and check in with participants. Use the emoji’s to see how everyone is feeling.>

Now we’re moving onto a section where I’m going to give you a scenario and then ask you some questions about it.

**Scenario 1**

**(Blood test for fatigue)**

You are seeing your GP, as you haven’t been for a check-up in a while and you’re feeling pretty run down. You explain to the doctor that you get plenty of sleep, yet when you wake up, you are still feeling tired. The doctor asks you about your diet and exercise habits. You do eat pretty well most of the time and have a wrist pedometer that counts your steps. The doctor suggests you have some blood tests and lets you know there is a pathology nurse available next door.

1. In this scenario, what types of things might encourage you to ask questions about the diagnostic tests the doctor has ordered? (e.g. relationship with GP, time etc)
2. In this scenario, what types of things might discourage you to ask questions about the diagnostic tests the doctor has ordered? Probe for environment, relationship and type of doctor.
3. In this scenario describe your level of trust in the doctor to order the right tests for you.
4. PROMPT: Do you think they ever get it wrong? How often? In what circumstances or scenario? Why might they get it wrong?
5. What do you think the types of diagnostic tests ordered in this scenario tell your doctor?

PROMPT. If you needed to make a change in your lifestyle (for example cutting out a food you love), do you think the test result would be enough to persuade you to do this?

1. In this scenario what are some of things you consider or the thought process you go through to decide if you will have the test?

Consider asking about if they can see any downsides to having an unnecessary blood test?

<5 min move break>

**Scenario 2**

You are seeing your GP today as you’ve got a sore lower back that has been bothering you for a couple of weeks. It’s interfering with your daily activities and your sleep. You’ve tried heat packs, cold packs, and a course of anti-inflammatories the pharmacist recommended. Nothing has worked and you’re a bit fed up with it. You’re pretty sure you need some diagnostic imaging done, but your doctor is refusing to order the test.

1. Thinking about this scenario, if you’ve been in a similar situation how did you handle it?
2. PROMPT would you handle it the same way again? different? how?
3. PROMPT if you haven’t been in this scenario imagine what you might you do? e.g. ask questions etc. How might you handle it? why/why not/how
4. In this scenario, why do you think the doctor is refusing to order a diagnostic image?
5. PROMPT How would this make you feel?
6. PROMPT What would help you understand the reason for the doctor’s refusal of your request?
7. PROMPT Describe what your next steps might be? Probe fpr ask questions, go away and research, talk to friends/family, see a different doctor etc.
8. How important do you think it is, to always know the medical reason we have symptoms, like back pain? Why/why not
9. PROMPT How do you feel not knowing the reason for a symptom? What types of things do we tell ourselves? What strategies do we use to cope with this uncertainty?
10. PROMPT Even if we know the reason, how important is it to always act on a diagnosis?

<STOP and check in with participants. Use the emoji’s to see how everyone is feeling.>

That concludes my questions, but is there anything else anyone would like to add?
