# Supplementary material for: How Do Australians Manage Diagnostic Testing Risks? Focus Groups Linked to a Model of Behaviour Change
Source: Health Expect. 2024 Oct 2;27(5):e70038. doi: 10.1111/hex.70038 (PMC11447086; doi:10.1111/hex.70038)
Supplement: Supplementary file 3 — Supporting information. [file HEX-27-e70038-s002.docx]

| Appendix 3: Themes mapped to the COM-B model with explanations for judgements | | | | | | | | |
| --- | --- | --- | --- | --- | --- | --- | --- | --- |
| **Theme** | **Which COM-B domain does theme map to? *, **** | | | | | | **Explanation for COM-B mapping** | **For managing test risks, is it?**  **- enabler**  **- barrier**  **- both**  **- depends** |
|  | **C: Phy.** | **C: Psy.** | **O: Phs.** | **O: Soc.** | **M: Ref.** | **M: Aut.** |  |  |
| 3.1.1. We’re informed patients | Theme primarily relates to psychological capability, as is evidenced by its two subthemes below. | | | | | | |  |
| *3.1.1.1. Proficient information appraisers* | **✓** | **✓** | **✓** |  | **✓** |  | Explicitly describes capability to find, select, appraise, and use information about testing choices and risks.  Motivation to seek out information is implied within the subtheme (e.g. motivation to do individual research), as is physical capability and opportunity to do so (e.g. access to resources). | ***Enabler***: can and will appraise information. |
| *3.1.1.2. Clinical judgements about testing scenarios* | **✓** | **✓** |  |  | **✓** | **✓** | Explicitly describes capability because it captures that participants felt able to make critical judgements about the testing choice scenarios.  Motivation and physical capability are implied in participants making self-initiated critical judgements about the scenarios. | ***Enabler***: can and will critically engage with test choices.  ***Depends/barrier:*** possible overconfidence about anaemia or sleep apnoea being likeliest causes of fatigue. |
| 3.1.2. Interpreting testing risks through the prism of experience | **✓** | **✓** |  |  | **✓** | **✓** | Reflective and automatic motivation are directly evidenced in participants drawing on experiences, both consciously and likely subconsciously, to interpret focus group questions about testing risks and benefits.  Capability is implied in participant references to past experiences and their analysis of these experiences to consider testing choices. | ***Depends***: experiences will shape understanding and willingness to manage test risks. |
| 3.1.3. Information needs when facing testing choices | Theme primarily captures motivation for seeking knowledge about tests in healthcare consultations when facing testing choices. | | | | | | |  |
| *3.1.3.1. Participants sought to understand risks and benefits* |  | **✓** | **✓** | **✓** | **✓** | **✓** | Reflective motivation is captured in overt perceptions that risks, benefits and/or rationale for a test need to be understood and negotiated in SDM. Automatic motivation is evident in some of the comments explaining beliefs about testing, such as the perceived benefits of catching disease early using tests.  Participant descriptions of successfully undertaking SDM about risk/benefit/rationale when presented with tests imply psychological capability (can engage in SDM) and both domains of opportunity (physically and socially have opportunity to engage in SDM). | ***Enabler***: willing to engage in SDM about test risks (and benefits) and the rationale for testing, and appear to consider themselves able to do so. |
| *3.1.3.2. Make test decisions with patients* |  | **✓** | **✓** | **✓** | **✓** | **✓** |  |  |
| 3.1.4. Relationships with doctors impact testing choices in complex ways |  | **✓** | **✓** | **✓** | **✓** | **✓** | Psychological capability is directly evidenced in participant descriptions of how they negotiated relationships with their doctors when considering test choices. Physical and social opportunity are directly evidenced in participant descriptions of SDM about testing in healthcare interactions. Reflective motivation is directly evident in descriptions about why participants want certain behaviours/discussions with their healthcare workers in relation to testing.  Subconscious beliefs and emotions such as trust, concern, fear, are implied in those comments. | ***Depends:*** if trust doctor, more capable but less motivated to engage in SDM about risks.  If distrust doctor, less capable but more motivated to engage in SDM about risks. |
| 3.1.5. Importance of asserting needs and preferences in testing decisions | **✓** | **✓** |  | **✓** | **✓** | **✓** | Capability is directly evidenced in participants’ descriptions of their self-efficacy and interpersonal skills to negotiate clinical encounters about testing and test risks. Reflective motivation is directly evidenced in their descriptions of desire to understand their testing choices and risks, and assert their preferences in clinical encounters.  Belief systems are implied (automatic motivation), as is permissibility to assert needs and preferences (social opportunity). | ***Enabler***: motivated and capable in asserting preferences in testing decisions.  ***Barrier***: limited understanding of test risks, evidence of broad desire for tests, willingness to change doctor if desire unmet. May lead to negation of testing risks. |
| 3.1.6. Structural factors influencing testing choices | **✓** |  | **✓** |  | **✓** |  | Physical opportunity is directly evidenced in descriptions of material constraints to testing choices (income, accessibility).  Physical capability and reflective motivation are implied in participants’ descriptions of their ability and willingness to overcome material constraints. | ***Depends***: cost, accessibility, structural factor considerations will variously promote/hinder considering and negotiating test risks. |
| *** **✓ *Black underlined*** *tick symbol = direct evidence of COM-B domain in theme;* **✓ *grey tick symbol*** *= implied evidence of COM-B domain in theme.* | | | | | | | | |
| *** COM-B refers to Capability, Opportunity and Motivation and their influence on Behaviour. Each of C, O, M have two subdomains. C: physical and psychological; O: physical and social; M: reflective and automatic.* | | | | | | | | |
